# Conceptualizing the Contextual Dynamics of Safety Climate and Safety Culture Research: A Comparative Scientometric Analysis

**DOI:** 10.3390/ijerph19020813

**Published:** 2022-01-12

**Authors:** Jie Li, Floris Goerlandt, Karolien van Nunen, Koen Ponnet, Genserik Reniers

**Affiliations:** 1 National Science Library, Chinese Academy of Sciences, Beijing 100190, China; lijie_jerry@126.com; 2 State Key Laboratory of Explosion Science and Technology, Beijing Institute of Technology, Beijing 100081, China; 3 College of Safety Science & Engineering, Liaoning Technical University, Huludao 125105, China; 4 Department of Industrial Engineering, Dalhousie University, Halifax, NS B3H 4R2, Canada; 5 Research Chair Vandeputte, University of Antwerp, 2000 Antwerp, Belgium; K.L.L.vanNunen@tudelft.nl; 6 Safety and Security Science, Faculty of Technology, Policy and Management, Delft University of Technology, 2600 AA Delft, The Netherlands; 7 Faculty of Political and Social Sciences, imec-mict-Ghent University, 9000 Ghent, Belgium; Koen.Ponnet@ugent.be; 8 Antwerp Research Group on Safety and Security (ARGoSS), Faculty of Applied Economics, University of Antwerp, 2000 Antwerp, Belgium; 9 Centre for Economics and Corporate Sustainability (CEDON), KU Leuven, 1000 Brussels, Belgium

**Keywords:** safety climate, safety culture, scientometrics, bibliometrics, VOSviewer, CiteSpace

## Abstract

Safety climate and safety culture are important research domains in risk and safety science, and various industry and service sectors show significant interest in, and commitment to, applying its concepts, theories, and methods to enhance organizational safety performance. Despite the large body of literature on these topics, there are disagreements about the scope and focus of these concepts, and there is a lack of systematic understanding of their development patterns and the knowledge domains on which these are built. This article presents a comparative analysis of the literature focusing on safety climate and safety culture, using various scientometric analysis approaches and tools. General development patterns are identified, including the publication trends, in terms of temporal and geographical activity, the science domains in which safety culture and safety climate research occurs, and the scientific domains and articles that have primarily influenced their respective development. It is found that the safety culture and safety climate domains show strong similarities, e.g., in dominant application domains and frequently occurring terms. However, safety culture research attracts comparatively more attention from other scientific domains, and the research domains rely on partially different knowledge bases. In particular, while measurement plays a role in both domains, the results suggest that safety climate research focuses comparatively more on the development and validation of questionnaires and surveys in particular organizational contexts, whereas safety culture research appears to relate these measurements to wider organizational features and management mechanisms. Finally, various directions for future research are identified based on the obtained results.

## 1. Introduction

Safety climate and safety culture are both widely used concepts in the safety science community, and there is a continued interest in applying their associated theories and methods in sectors such as the nuclear and petrochemical industries, rail transport and aviation, and healthcare [1,2,3]. The concept “safety climate” was first introduced by Zohar [4], while the concept “safety culture” was first reported by the International Nuclear Safety Advisory Group in their report [5] following the Chernobyl disaster in 1986. Safety climate as a concept is therefore older than the concept of safety culture.

Despite the significant interest in the concepts, theories, and methods of the safety culture and climate research domain, there are various interpretations regarding the conceptual contents of each and their interrelationship [6], with debates continuing to the present day [1,7]. For instance, some scholars (e.g., [8]) consider safety climate as a constituent part of safety culture, where the former concerns shared perceptions of safety influenced by socio-organizational aspects such as leadership, organizational trust, management commitment, and transparent communication. In this conceptualization, safety culture is a wider concept which dynamically and cyclically relates safety climate to the psychological attitudes, perceptions, and behaviors of individuals within the organization, and to the technological, educational, procedural, and bureaucratic features of organizational management. In contrast, other scholars (e.g., [7], citing [9]), consider these concepts in a non-hierarchical way, where safety culture creates the conditions in which a safety climate appears. Safety culture hence falls within the remit of senior top management, whereas safety climate falls within the area of responsibility of lower and middle management. For further insights into the debates about the conceptual contents of safety culture versus safety climate, see, e.g., [10].

Several review articles have been published to summarize the ideas, developments, and practices, reflect on knowledge gaps, and propose future research directions for safety culture and safety climate research. A selection of review articles is outlined below.

Guldenmund [6] reviewed safety culture and climate literature within the framework of organizational culture as described by Schein in their paper [11], distinguishing basic assumptions, espoused values, and artefacts. Rall and Dieckmann [12] reviewed the literature on safety culture and high-reliability organizations (HROs), and combined these topics with the principles of crisis resource management in a context of patient safety. Powell et al. [13] reviewed safety culture in food safety, and discussed its concepts in the context of three case studies, focusing on the importance of proactive risk management, continuous learning, communication systems, and blame-free interactions with customers. O’Connor et al. [14] focused on safety climate in commercial and military aviation, and reviewed questionnaires to measure the construct. Henriqson et al. [15] reviewed the safety culture literature from a discursive perspective, and highlighted four effects: a focused organizational culture implying normative homogeneity, an enforcement of workers’ safety behavior, a political control of organizations, and a governmentality connecting individual conduct with organizational norms. Hessels and Larson [16] reviewed the relation between patient safety culture and adherence to standard precaution procedures, with regard to healthcare worker safety. Nyarugwe et al. [17] reviewed food safety culture research, finding that organizational and administrative characteristics, technical resources, employee and group characteristics, and national culture were among the elements to focus on in understanding safety culture. Manser et al. [18] reviewed instruments available in German-speaking countries to measure safety climate in healthcare. Goncalves and Waterson [19] reviewed the use of maturity models in safety culture, finding that these are increasingly used but that there is typically little focus on the reliability and validity of the findings. Vanconcelos et al. [20] reviewed the literature on instruments to measure safety culture in primary healthcare, identifying three instruments with acceptable psychometric properties. Vierendeels et al. [8] reviewed the safety culture and safety climate literature and proposed an integrated conceptual framework for safety culture. Nævestad et al. [21] reviewed aspects of the regulation of safety culture, addressing, inter alia, what types of regulatory efforts are made, what strategies regulators apply to influence safety culture, and experiences with and results of these strategies. Wang and Wu [22] reviewed the development of safety culture research in China, highlighting its achievements and future research needs. Yorio et al. [23] reviewed organizational safety culture and national culture, and theorized that organizational beliefs, assumptions, and values reflect the national culture in which the organization is embedded.

Within the large volume of safety culture research, scientometric analysis has been used by van Nunen et al. [24] to obtain high-level insights into the research domain, identifying author and institutional collaboration networks, influential journals and articles, and co-citation networks. Scientometric analysis is a method of using quantitative and statistical measures of reference and citation information from the published literature to obtain insights into structural and narrative patterns and trends in a given domain, often using visual techniques (see, e.g., Li et al. [25] and Section 2.2).

Unlike narrative literature review approaches [26], scientometric analysis techniques use quantitative and statistical methods to detect high-level patterns in the research domain. Scientometrics is thus better suited to providing insights into the development of a body of research, including narrative clusters, trends in topics addressed, highly influential articles, and knowledge communities involved in its development. In the safety sciences, several scientometric mapping studies have already been performed to gain broad insights into subdomains of the research field. For example, analyses have identified the safety science outputs and topics of core safety science journals [27,28]. More recently, a high-level analysis of the development of the journal *Safety Science* was presented [29], where patterns and trends in emergency evacuation safety were identified [30], building information modeling in construction safety was scientometrically analyzed [31], and other work addressed resilient healthcare [32] and university laboratory safety [33]. For a recent overview of safety-related scientometric analysis mapping applications, see [25].

Given the somewhat contentious relationship between safety culture and safety climate as indicated above, it is worthwhile to better understand the differences and similarities between the knowledge domains concerned with each of these concepts. In this study, a comparative analysis of the literature focusing on safety culture and safety climate was performed. Using scientometric analysis methods, insights were obtained into the development trends and structural patterns of these knowledge domains. Knowledge was also obtained about its influencing science domains, its primary contributing scientific communities, and which articles from outside these fields have impacted its development. These insights contribute to the discussion on the distinctions between safety climate and safety culture, while also helping academics understand the major trends and contributing knowledge domains and articles to the safety climate and safety culture literature. Based on this, several directions for future research directions are identified.

The remainder of this article is organized as follows. Section 2 outlines the data sources and the database construction process, and briefly introduces the scientometric analysis and mapping methods. Section 3 shows the results, including: statistical comparison of the temporal, geographical, and publication trends in safety climate and safety culture domains; the journals and scientific categories in which the research domains are located and on which they draw knowledge; a topic comparison providing insight into the keyword patterns associated with each domain; and a citation analysis to identify publication patterns and influential articles supporting the knowledge domains. Section 4 provides a discussion on the findings, reflects on the limitations of the work, and outlines future research directions. Section 5 concludes.

## 2. Data and Methods

### 2.1. Data Source

Several bibliographic databases can be used to collect scientific literature. Among these databases, the Web of Science Core Collection (WOSCC) is one of most widely used databases for scientific literature. WOSCC not only has a long history and a variety of data but it also has scientific literature data with indexing of the highest quality [25]. In the present research, papers (including “article” and “review” document types) published in scientific journals and indexed in Web of Science (WOS) in the “Science Citation Index Expanded (SCIE)” and “Social Sciences Citation Index (SSCI)”, were searched for and downloaded.

In order to search for the most relevant records of “safety climate” and “safety culture” literature included in the WOSCC database, the “title search strategy” was applied. This search strategy means that the records were searched, and items were downloaded if the term “safety climate” or “safety culture” appeared in the title. This focused search strategy was chosen to ensure that the work focused specifically on the intended concepts, rather than perhaps having a more peripheral relation to safety culture or safety climate, which may have been the case if, for instance, the title, keywords, and abstracts were targeted in the search.

**Figure 1:**
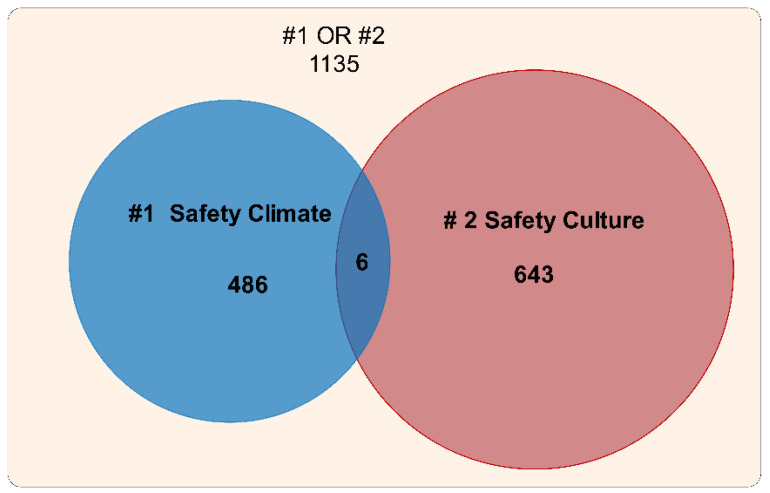
Venn diagram of papers in SSCI and SCIE (August 2019) including “safety climate” or “safety culture” in the title.

The initial dataset was collected in October 2018, and the data were subsequently updated so that the time span covered included the full year of 2018. Hence, the time span considered in the search included all publications up to and including 2018. A Venn diagram describing the collected datasets is shown in Figure 1. There were 1135 papers that included “safety climate” or “safety culture” in the title. A total of 649 of these 1135 papers were records related to “safety culture”, while 492 records were related to “safety climate”. In 6 papers, “safety climate” and “safety culture” were both mentioned in the title. Consequently, there were 486 records linked only to “safety climate” and 643 records linked only to “safety culture” research.

### 2.2. Methods

**Figure 2:**
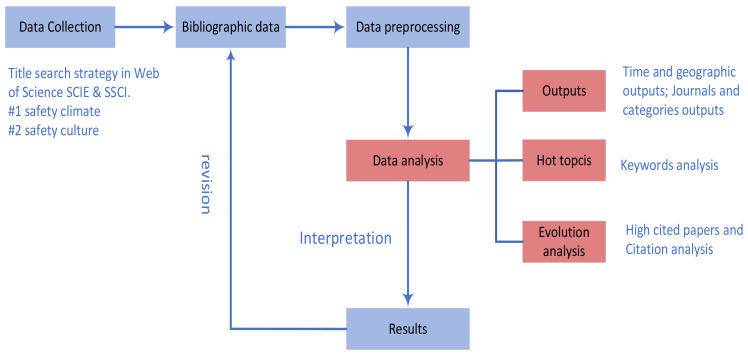
Flowchart of the scientometric analysis for the comparison of safety climate and safety culture publications.

In order to compare the scientific publications on safety climate and safety culture, the collected data were analyzed and compared using scientometric mapping methods, i.e., quantitative analysis methods and visualization techniques were applied to the bibliographic data of the relevant publications. A summary of the research process is shown in Figure 2.

First, the data were collected using the Web of Science SCIE and SSCI database, as explained in Section 2.1. From this dataset, the bibliographic data were obtained by extracting the data fields of all articles in the dataset in text file format. These data were subsequently preprocessed, which was necessary to increase the accuracy and reliability of the results. This included checking whether the data format met the requirements of the research purposes as, for instance, some software analysis tools may require all text to be in lowercase, whereas database entries may include capitals. Another example of data cleaning, which was usually executed in a revision loop after an initial data analysis was performed, as indicated in Figure 2, was the disambiguation of author or institution names (see, e.g., [34] for an approach to this issue).

The data analysis stage in this study consisted of the application of diverse scientometric mapping tools to perform the following: analyzing outputs from temporal and geographical perspectives; identifying patterns in the journals and the scientific categories in which safety climate and safety culture research is situated. and from which of these their knowledge base is drawn; analyzing keywords associated with these domains; and identifying highly impactful articles internal and external to the research domains and the dependencies between them. HistCite software [35] was used to identify the major institutions that have made contributions to the published literature and to analyze the countries/regions of origin. The journals, categories, keywords, and citation networks were analyzed using CiteSpace [36], the Loet Leydesdorff disciplinary overlay toolkit [37], VOSviewer [38], and CitNetExplorer [39]. CiteSpace is a specific tool which can be used to create the journal dual-overlay maps and to show the major citation connections between citing journals and cited journals. The Loet Leydesdorff disciplinary overlay toolkit was used to generate the category overlay profile and measure the diversity of the research. VOSviewer software was also used to analyze the co-keywords network, and to obtain the topic clusters and trends of the research. CitNetExplorer was applied to construct the direct citation networks, showing the document clusters and evolution of the research. For an introductory overview of these tools, the outputs they generate, and their underlying methods, see [25].

Finally, the results were interpreted. This relied on the understanding and insights of the analyst to detect, describe, and explain the patterns observed in the visual mappings of the literature. As explained above, if needed, an iteration was used to obtain accurate and reliable results, revising bibliographic data, e.g., to disambiguate keywords, which may obfuscate patterns or otherwise bias the results.

## 3. Results

In this section, the results of the scientometric analyses are shown, providing the insights into the stated aims of the article, as described at the end of the Introduction (Section 1).

### 3.1. Outputs Comparison Analysis

#### 3.1.1. Publication Trends Analysis

As mentioned in Section 2.1, in total, 492 records related to safety climate and 649 records related to safety culture were obtained from WOS. The basic information on the bibliographic data is presented in Table 1. The first article introducing safety climate was written by Zohar in 1980 [4], while the first three scientific papers concerning safety culture were published in 1991 [40,41,42]. There were 478 articles and 14 review papers on safety climate, and 622 articles and 27 review papers on safety culture research. Papers from the safety climate dataset had a larger number of “keywords” than those from the safety culture dataset. In contrast, articles from the safety culture dataset had a larger number of associated “authors”, and “journals”.

**Figure 3:**
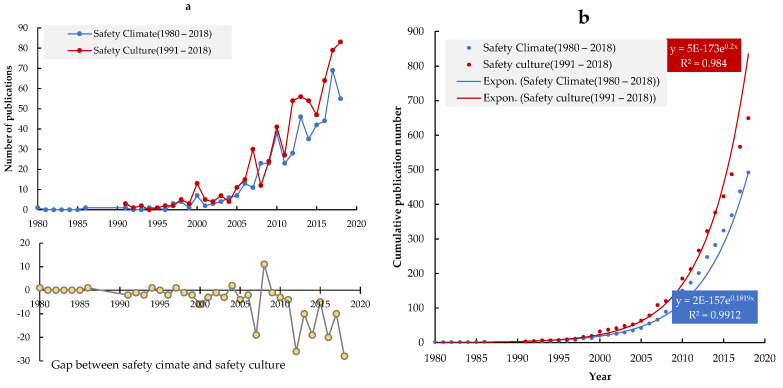
Trends in publication output of papers on safety climate and safety culture from 1980 to 2018. (**a**) Annual trends of the safety climate and safety culture publications, (**b**) Cumulative publication trends of safety climate and safety culture research.

As can be seen in Figure 3a, the notion of “safety climate” was first introduced in 1980 [4]. Later, following the Chernobyl disaster in 1986, the concept of “safety culture” entered the field of safety research. Before 2000, both safety climate and safety culture were the focus of only a few scientific publications. As a new concept, researchers needed to discover the scope and focus of the research domain and explore its fundamental concepts, which requires time.

The year 2000 was an important year for safety climate and culture research, because from then onwards the number of papers in the two areas increased rapidly. The trends reflect the fact that research on safety climate and culture became more mature in the field of safety research. Comparing publications on safety climate with publications on safety culture before and after 2000, it was found that before 2010, there was no significant gap between the two. However, after 2010, the number of articles on safety culture exceeded the number of safety climate papers. The cumulative number of publications on safety climate and safety culture is shown on the right side of Figure 3b. The total volume of the safety climate and safety culture literature follows an exponential increase. Compared with safety climate, the volume of safety culture publications has grown faster.

#### 3.1.2. Geographic Distribution Analysis

The geographic distribution of global safety climate and safety culture publications was analyzed. The top 10 most productive countries/regions in safety climate and safety culture research (in absolute terms) are listed in Table 2. The most productive countries/regions in the field of safety culture research also appear in the list of most productive countries/regions in the field of safety climate research.

Researchers from the USA published the highest number of papers on safety climate (179 papers in total), followed by Australia (n = 84), the UK (n = 51), and China (n = 41). The safety climate output of the other countries/regions were all below 30 papers. Among these countries/regions, the “total number of citations (TNC)” and the “citations per paper (CPP)” are indicators of the total impact and average impact of the papers in each country/region. As shown in Table 2, the USA (TNC = 6895) had the highest number of total citations in safety climate research, followed by Israel (TNC = 3690), Australia (TNC = 3501), and the UK (TNC = 3491). The citations per paper, however, revealed that Israel, with 141.92, was ranked in first place, followed by the UK (CPP = 68.45), and Canada (CPP = 57.74). In safety culture research, the USA was the most productive country with 174 papers, followed by the UK (n = 74), Australia (n = 37), Netherlands (n = 37), China (n = 33), and Canada (n = 31). The total number of citations revealed that the USA, with 3902 citations, was ranked first among the countries/regions, followed by the UK (TNC = 3101). Although only 37 papers originated from the Netherlands, these received a total of 1644 citations, and they also had the highest number of citations per paper (CPP = 44.43) in safety culture research. The UK also performed well, with 41.91 citations per paper.

Comparing the highly productive countries/regions in safety culture and safety climate with each other, Israel, Sweden, and Brazil stand out. Israel and Sweden were highly productive in the domain of safety climate but not in the domain of safety culture, while Brazil was highly productive in safety culture but not in safety climate.

As can be seen in the list of the top 10 productive institutions (see Table 3), Harvard University was the most productive university with 23 papers in safety climate research, followed by the Liberty Mutual Research Institute for Safety (n = 19), Technion–Israel Institute of Technology (n = 18), University of South Australia (n = 17), and Queensland University of Technology (n = 16). Technion–Israel Institute of Technology had the highest total number of citations (TNC = 3601) and the highest citation rate per paper (CPP = 200.06). The University of Aberdeen also had a high impact, with a total number of 1697 citations and 141.42 citations per paper. The University of Pittsburgh was the most productive institution in safety culture research with 14 papers, followed by Vrije University Amsterdam Medical Center (n = 10), Johns Hopkins University (n = 9), University of Michigan (n = 9), and Vrije University Amsterdam (n = 9). The total number of citations of the University of Pittsburgh was 550, with this institution ranked in first position; however, it ranked fifth in terms of citations per paper, with a value of 39.29. Griffith University published 8 papers with a total number of 544 citations, and was ranked in first position for citations per paper (CPP = 68.00). The Westat Corporation (TCN = 490, CPP = 61.25) and Johns Hopkins University (TCN = 547, CPP = 60.78) also performed better than other institutions.

Comparing the highly productive institutions with each other in the domain of safety climate and safety culture, the analyses revealed that the distribution of highly productive institutions was significantly different depending on the countries/regions. Only one institution (University of Manchester) appeared on both sides of the highly productive institutions list. However, as seen in Table 2, the most productive countries/regions had a higher overlap between safety climate and safety culture research, implying that within highly productive countries/regions, different institutions focus on safety culture and safety climate.

#### 3.1.3. Journals Output Distribution

Journals dual-overlay analysis was introduced by Chen and Leydesdorff [36]. It presents the distribution of citing and cited journals, as well as the citation links between them, on a global journal science map. This global journal science map shows all journals in all scientific domains, grouped in clusters associated with major fields of science, essentially showing the structure of the sciences. The clusters of the journals were generalized by Blondel and labeled with the log-likelihood ratio (LLR).

**Figure 4:**
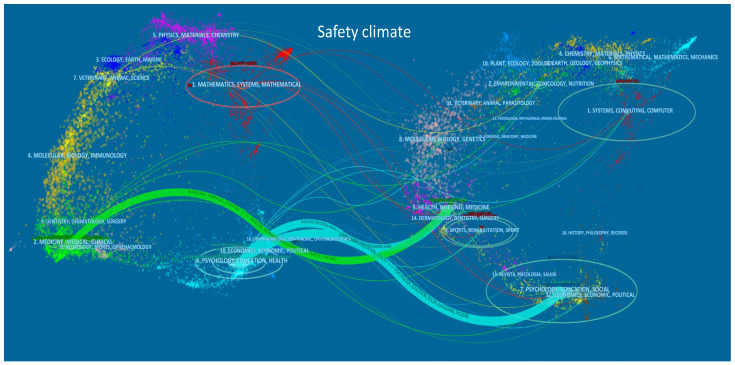
Dual map overlay of safety climate publications (generated using CiteSpace, see Section 2.2 for references). Note: The left-side clusters represent citing domains while the right-side clusters are cited domains; 154 citing journals and 4920 cited journals were mapped.

**Figure 5:**
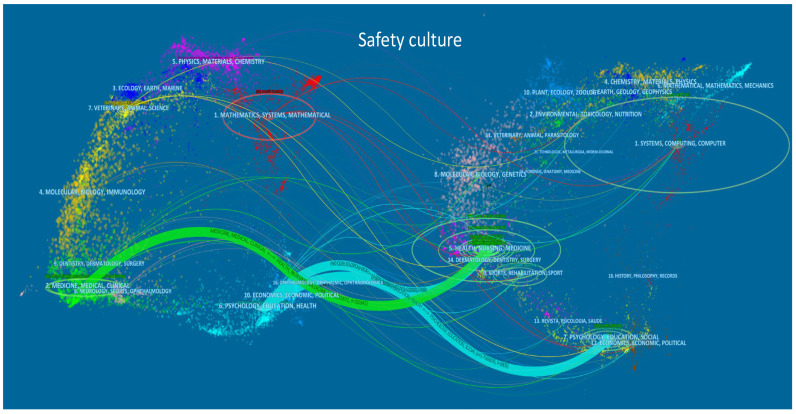
Dual-overlay map of safety culture publications (generated using CiteSpace, see Section 2.2 for references). Note: the left-side clusters represent citing domains while the right-side clusters are cited domains; 273 citing journals and 6842 cited journals were mapped.

Figure 4 and Figure 5 show the journals dual-overlay map of safety climate and culture research. The size of the oval on the left side shows the number of papers and authors in the citing journals, and the right side shows the number of citations of the cited journal. The thickness of the lines is proportional to a z-score-scaled frequency of citation [43], and indicates the connection strength between the domains of science within the analysis, i.e., safety climate in Figure 4 and safety culture in Figure 5. Hence, the dual-overlay map allows the identification of patterns showing how specific domains (citing journals) are influenced by other domains (cited journals). Three mainstream citation paths can be identified in safety climate and safety culture research. Detailed information on these is summarized in Table 4.

The relationships between the citing journals and cited journals were sorted by the z-score in descending order. It was found that the safety climate and safety culture research domains had similar citation trajectories. Safety climate and safety culture research was mainly published in “psychology, education, health” and “medicine, medical, clinical” types of journals, and were also influenced by similar domains (“psychology, education, social” and “health, nursing, medicine”). This shows that the knowledge base for both safety climate and safety culture is most intensely researched in the healthcare domain, and that psychological and social science concepts, theories, and methods are predominantly associated with these concepts.

The outputs and citations of the journals were obtained using VOSviewer, and the results are shown in Table 5. The left side shows that the highly productive journals for publishing safety climate papers were *Safety Science* (58), *Accident Analysis and Prevention* (44), *Journal of Safety Research* (30), *BMJ Quality & Safety* (14), and *Journal of Applied Psychology* (14). Highly productive journals for publishing safety culture papers were *Safety Science* (59), *BMC Health Services Research* (35), *International Journal for Quality in Health Care* (23), *BMJ Quality & Safety* (22), and *Journal of Patient Safety* (17). *Safety Science* was ranked in first place for both safety climate and safety culture research, reflecting the fact that *Safety Science* is a key journal in this field of research.

Further analyzing these highly productive journals, it appeared that safety climate papers were primarily published in accident-, quality-, and psychology-related journals. Safety culture papers were more often published in health- and patient-related journals. These highly cited journals can be regarded as the “intellectual bases” supporting safety climate and safety culture research. In safety climate research, the psychology-, safety-, and accident-related journals played a core role and received more citations than other journals. In safety culture research, safety- and health-related journals were the core intellectual bases and received higher numbers of citations.

**Figure 6:**
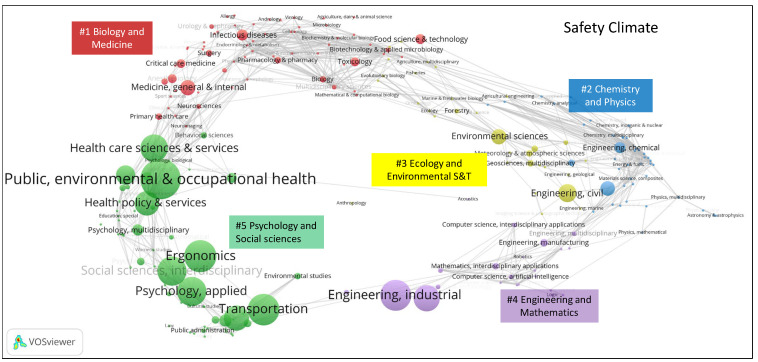
Location of Web of Science categories for safety climate publications in the global science overlay map (generated using the Loet Leydesdorff disciplinary overlay toolkit and visualized using VOSviewer; see Section 2.2 for references).

**Figure 7:**
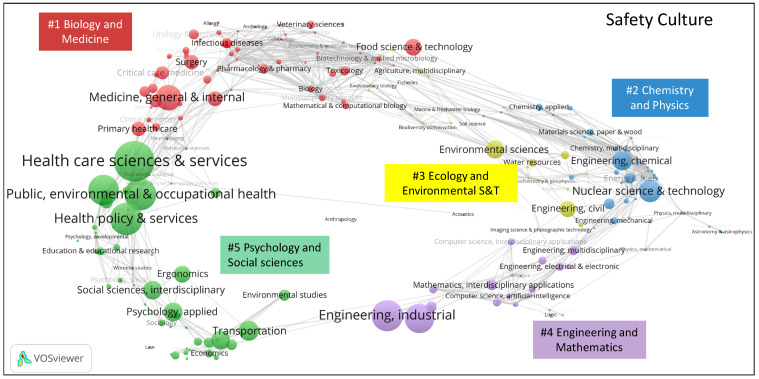
Location of Web of Science categories for safety culture publications in the global science overlay map (generated using the Loet Leydesdorff disciplinary overlay toolkit and visualized using VOSviewer; see Section 2.2 for references).

In Web of Science, journals are assigned to at least one of the 248 categories from the Science Citation Index Expanded and Social Science Citation Index. The distributions of these Web of Science categories assigned to the safety culture and safety climate publications in the global science map are shown in Figure 6 and Figure 7. Safety climate papers have been published in 35 Web of Science categories, while safety culture papers have been published in 75 Web of Science categories.

Category overlay maps for safety culture and safety climate research were created using the Stirling–Rao diversity measure. This is a measure that takes into account the variety, balance, and disparity of categories in a distribution [44]. The Stirling–Rao diversity of safety climate publications is 0.684, and the value is 0.759 for safety culture publications. This means that safety culture research is characterized by a higher diversity than safety climate research. Hence, safety climate research has a narrower domain of covered science categories.

Carvey et al. [37] clustered the global science overlay map into five clusters: #1 biology and medicine, #2 chemistry and physics, #3 ecology and environmental S&T, #4 engineering and mathematics, and #5 psychology and social sciences. Safety culture and safety climate publications are both mainly located in the “psychology and social sciences” area. The categories of “nursing”, “medicine”, “nuclear”, and “chemical” have a higher concentration in safety culture publications. The categories “ergonomics”, “social sciences interdisciplinary”, “psychology”, and “management” are more strongly associated with safety climate publications.

### 3.2. Focus Topics Comparison Analysis

Keywords selected by a paper’s authors are commonly used to highlight the key topics addressed and to give an impression of the content of the paper. Keywords displaying a high frequency in all publications on a certain topic can be regarded as significant focus topics in the considered research domain. In this work, a keywords co-occurrence network was created using VOSviewer, not only to show the keywords with high occurrence frequency but also to obtain insight in the co-occurrence of keywords in the same paper. Keywords with an occurrence frequency of two or more were selected to construct the keywords co-occurrence network. A total of 126 out of 914 keywords met the threshold in safety climate research and 100 out of 1066 in safety culture research.

**Figure 8:**
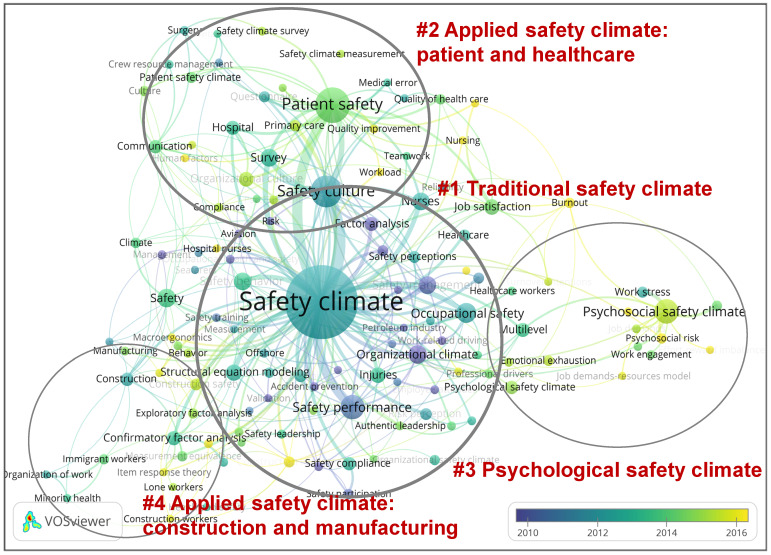
Clusters and average occurrence years of author keywords in safety climate research (generated using VOSviewer; see Section 2.2 for references).

**Figure 9:**
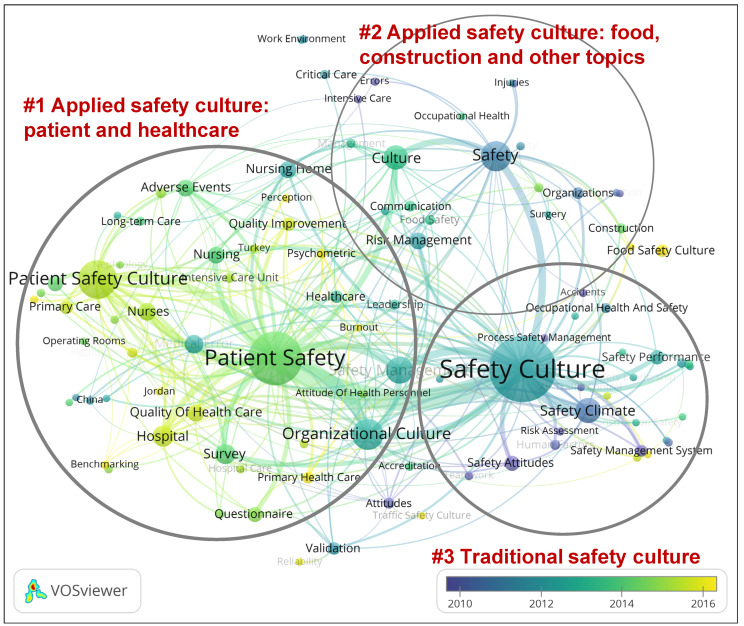
Clusters and average occurrence years of author keywords in safety culture research (generated using VOSviewer; see Section 2.2 for references).

The keywords co-occurrence network for safety climate research is shown in Figure 8, and that for safety culture research is shown in Figure 9. The most frequently occurring keywords in the safety climate and safety culture research datasets are listed in Table 6. The node size of the keywords is associated with the keyword occurrences. The larger the node size, the higher the occurrence frequency. In this analysis, the number of occurrences of a keyword is equal to the number of papers in which the keyword appeared. The thickness of the links demonstrates the co-occurrence strength between two keywords. The more often two keywords co-occurred, the more relevant were those keywords in determining narrative clusters in the research domains. The co-occurrence network of the keywords was clustered using the modularity method in VOSviewer, and nodes are marked with keywords and circles in Figure 8 and Figure 9. The color of each node indicates the average publication year of the keywords in the dataset, showing the temporal evolution of topics in the research field.

In safety climate research, the analysis of highly occurring keywords showed that “patient safety”, “safety culture”, “psychosocial safety climate”, “safety performance”, “occupational safety”, and “organizational climate” were amongst the primary hot topics. Furthermore, “patient safety”, “patient safety culture”, “organizational culture”, “safety management“, and “safety climate” were the hot topics in safety culture research.

Four clusters can be identified in safety climate research and three clusters in safety culture research, with the center of each cluster marked with a circle in Figure 8 and Figure 9. In safety climate research, Cluster #1, traditional safety climate research, is in the center of the keywords map. The topics in this cluster have a lower average year of publication, i.e., this is a relatively older cluster. “Safety culture”, “safety performance”, “occupational safety”, “organizational climate”, “safety behavior”, and “safety management” are frequently occurring keywords in the traditional safety culture group. Other clusters mostly represent major areas of applied safety climate research. For example, Cluster #2 concerns the safety climate in patient and healthcare. Cluster #3 concerns psychological safety climates (PSCs) and is defined as policies, practices, and procedures for the protection of workers’ psychological health and safety [45], which is a comparatively newer area of activity within safety climate research. Cluster #4 is a small group of applied safety climate research topics within the construction and manufacturing industries. The keyword clusters of safety culture were clustered into three main groups of topics. Cluster #1 is traditional safety culture research and is located on the right side of the network, with the keywords’ average publication year close to 2010. “Safety climate”, “safety attitudes”, and “safety performance” are frequently occurring keywords in the traditional safety climate cluster, showing the most important topics in safety culture research.

Comparing safety climate and safety culture keywords showed that both domains had the same interest in “patient safety”, “safety performance”, “safety management”, “survey”, “organizational culture”, “hospital”, and “nurses”. There were a few differences between them: “psychosocial safety climate”, “organizational climate”, “safety behavior”, and “structural equation modeling” were highly occurring keywords in safety climate research, while “patient safety culture”, “medical error”, “nursing”, “adverse events”, and “nursing home” were highly occurring keywords in safety culture research. The keywords difference between safety climate and safety culture showed that Cluster #1, addressing traditional safety climate research, remained in the leading place in safety climate research, while Cluster #3, “traditional safety culture”, was overtaken in importance by patient and healthcare safety culture research. These changes reflect the fact that safety culture research has become active in practice, especially in the field of patient safety, as has also been shown in previous research [24].

Both keyword maps show that there are conceptual links between safety culture and safety climate, which are mostly located in Cluster #1 (traditional safety climate) and Cluster #3 (traditional safety culture). In safety climate research, the link to safety culture appears to be more narratively linked to applications within healthcare, as it overlaps with Cluster #2. In contrast, in safety culture research, safety climate is seen as a contributor to safety culture, along with other methodology-oriented keywords such as “safety management system”, “risk assessment”, and “human factors”.

### 3.3. Evolution of in Safety Climate and Safety Culture

Direct citation networks were created based on the local citation scores of citing papers in the safety climate and safety culture research domains. Citation links were constructed to show the evolution of safety climate and safety culture in the past few years. In the citation network, the nodes and labels stand for the citing papers on safety climate or safety culture. In order to avoid an overlap between citing paper labels, the label shows the last name of the first author, whereas the links indicate the citation relations between each citing paper.

**Figure 10:**
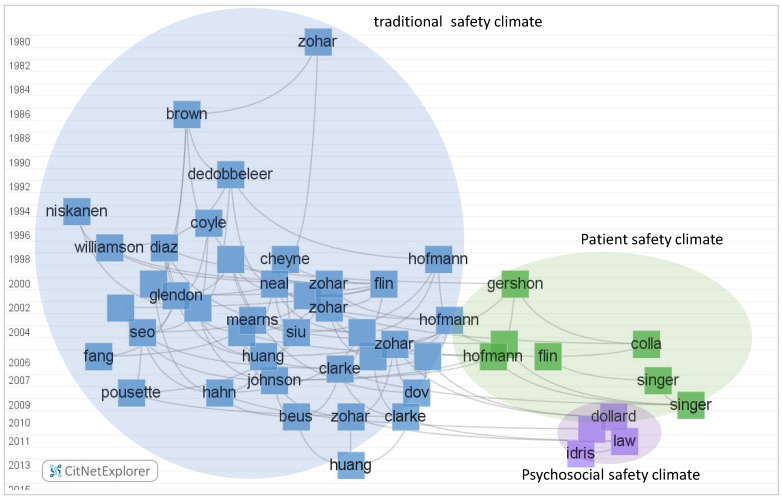
Direct citations network of safety climate publications: the top 50 most cited papers on safety climate are included in the current network. For details, see Supplementary Material Table S1. (Generated by CitNetExplorer; see Section 2.2 for references).

The direct citation links of the top 50 cited safety climate papers are shown in Figure 10. Three clusters in were found in the citation network of the safety climate domain, representing the “traditional safety climate”, “patient safety climate”, and “psychological safety climate” clusters of Figure 10. Based on the timeline of the citation links, it is clear that the “traditional safety climate” research cluster had a wider time span and is still an active research domain. The “patient safety climate” cluster, and especially the “psychological safety climate” cluster, were identified as comparatively more recently emerging research directions in safety climate research, confirming the findings of Figure 8.

**Figure 11:**
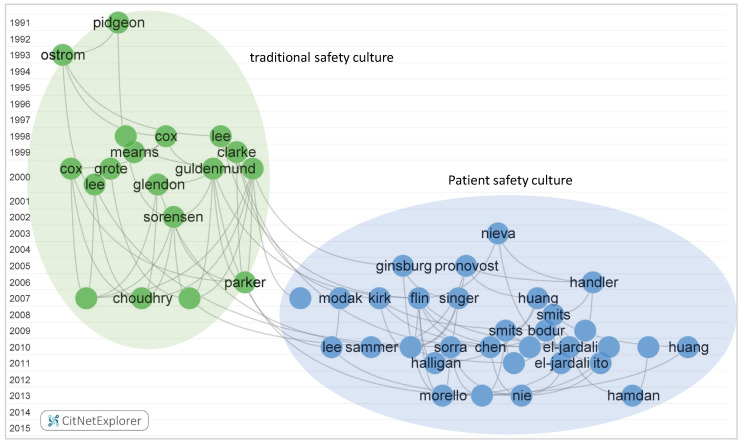
Direct citations network for safety culture publications: top 50 most highly cited papers on safety culture are included in the current network. For details. see Supplementary Material Table S2. (Generated by CitNetExplorer; see Section 2.2 for references).

**Figure 12:**
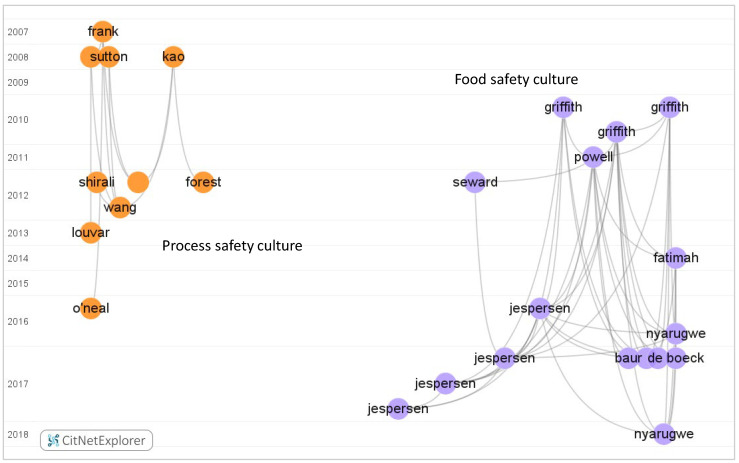
Direct citations network for process safety culture and food safety culture: 10 papers on process safety culture and 16 papers on food safety culture. See Supplementary Material Table S3 for details. (Generated by CitNetExplorer; see Section 2.2 for references).

Four clusters on safety culture can be discerned in the direct citation network, including “traditional safety culture”, “patient safety culture”, “food safety culture” and “process safety culture”. The top 50 most cited safety culture papers are shown in Figure 11. Two main clusters were revealed in the key citation network. The citations of food safety culture and process safety culture papers are not included in the top 50 most highly cited papers. However, the extracted citation network for process safety culture and food safety culture is shown in Figure 12. Detailed information on process and food safety culture articles can be found in Supplementary Material Table S3. In Figure 12, the “process safety culture” cluster of research is on the left side of the citation network and encompasses 10 papers. The “food safety culture” cluster is on the right side and includes 16 papers. The time span of process safety culture and food safety culture research demonstrates that there is more recent research in safety culture practice. Comparing these results with the findings of Figure 9 confirmed that “traditional safety culture” is an older area of activity, whereas “patient safety culture”, and especially “process safety culture” and “food safety culture” are more recent areas of activity and can be considered as new emerging research subdomains.

## 4. Discussion

The presented comparative analysis of the safety climate and safety culture research domains has revealed various trends, patterns, interrelationships, and differences. Rather than summarizing the findings, which is left to the Conclusions in Section 5, some methodological limitations and more general points of discussion are raised here. In addition, considering the high-level insights obtained through the scientometric mapping approach, several future research directions are identified.

It is important to stress that the analyses are merely descriptive of the research domains, identifying trends and patterns and highlighting major contributions, aiming to shed light on to what extent, and how, safety climate and safety culture overlap and differ. Hence, the current work does not intend to make statements about the correctness of the ideas within the research domain, the validity of the models or measurement tools, the usefulness of safety climate or culture in understanding organizational safety, or the effectiveness of these concepts in improving or managing organizational safety performance. Despite the large and growing volume of research, several authors have critiqued the concepts on a variety of grounds. Such critiques include, for instance, the lack of consensus on what exactly the concepts mean and the disparate views held in differing scientific disciplines on whether safety culture research is aimed primarily at understanding organizations (interpretivist) or as a mechanism to drive safety performance (functionalist) (see, e.g., [7]). Reiman and Rollenhagen [46] provide various other critiques, including the often simplistic treatment of organizations in safety culture theories, the monolithic treatment of culture, and the lack of specific consideration of the meaning of the concept of safety itself.

A related issue, which can be construed as a methodological limitation, is that the clustering of the research domains is based only on whether the title contains the textual string “safety climate” or “safety culture”. As shown in [24], if this search was performed covering the articles’ title, abstract, and keywords, many more hits would be found. In [24], 1789 articles were identified in such a search using “safety culture”, executed in December 2015, versus the 649 obtained in the present work, as outlined in Section 2.1. It is possible that the present findings concerning trends, patterns, similarities, and differences between safety climate and safety culture would be somewhat different if this type of wider search was performed and the resulting dataset analyzed. Nevertheless, the choice to include only the title in the search field was made to ensure that the articles indeed explicitly focused on safety climate and/or safety culture, so that these could be contrasted as sharply as possible. Including, e.g., the abstracts may have led to a dataset where safety culture was mentioned more peripherally, e.g., in the context of safety management systems or resilience research. Follow-up research could investigate the stability of the current findings in the light of this methodological uncertainty. A similar methodological issue is that our analysis, as described in Section 2.1, was based on the bibliometric data from both review papers and original research articles. It could be investigated whether similar results would be obtained if only data from original research articles were retained.

Furthermore, as indicated in the Introduction and elsewhere, the authors acknowledge the pervasive different interpretations of the contents, scope, focus, and purpose of the concepts of safety climate and safety culture. The search strategy and the scientometric analysis necessarily make an abstraction of these conceptual subtleties, and it is likely that not all authors in each research domain share a view on what these concepts exactly entail; they may even confuse one with the other. Hence, the analysis results should be understood as indicative and as providing insights into the community’s own understanding of its major ideas and purpose, which may contain contradictions and inconsistencies.

Another issue that is apparent from the results is that safety climate and safety culture, as subdomains of safety science, form rather diverse and multi-faceted conceptual and methodological clouds (see, e.g., Figure 8 and Figure 9). While these maps do not provide the specifics of particular methods in depth, they do signify that there is a large variety of models, methods, and frameworks, where safety climate and safety culture are linked to diverse theories, measuring tools, and mechanisms for understanding safety and/or managing safety performance. Along with a lack of convincing evidence for the validity, usefulness, and effectiveness of many of these [7], this leads to a “safety cloud” surrounding industrial organizations, i.e., a difficulty for companies in understanding and rationalizing the choice of models and methods for use within their organizational setting [47]. This highlights a need to place a further focus on method validation and on supporting organizations in selecting suitable methods.

Finally, some future research directions are identified. First, as found in Section 3.1 and Table 2, both the safety climate and safety culture domains are dominated by results from Western countries. Acknowledging the likely importance of the characteristics of national cultures to the safety culture in organizations, at least indirectly, as discussed in [23], an important area of future research will be to investigate safety climate and safety culture in non-Western cultures, and to make inter-cultural comparisons. Secondly, the results show that by far the most active application domain is healthcare, followed by the construction, process, and food industries. While there has been safety culture and safety climate research also in other industries, such as air and maritime transport [48,49], mining [50], and offshore workers [51], more research in these and other application domains could enrich the research field.

## 5. Conclusions

In this article, a scientometric analysis was presented, comparing the research domains focusing on safety climate and safety culture, which are both important areas of activity within safety science. Given previous and ongoing debates about the scope and focus of these concepts, and how these are related, it is useful to take a high-level view of these research domains to gain insight into what conceptual and methodological issues they focus on, what geographical areas and application sectors drive the research domains, and what knowledge bases their respective scientific communities rely on. Various scientometric analysis mapping methods were applied to provide insight into these questions. Furthermore, the most impactful articles in the respective research domains were identified, and their evolution patterns mapped.

The main findings were as follows. First, the literature in both research domains is growing at an exponential rate, with the safety culture body of literature being the larger, despite the historically delayed research activity. Most literature on both fields originates from Western countries, with the USA, Australia, and the UK in the top three for both domains. China is also an important and active contributor to both areas, and Brazil is a comparatively important contributor to the safety culture field but not to safety climate. In the top three countries, it is noteworthy that different institutions contribute to each research domain, with only the University of Manchester significantly contributing to both.

The safety climate and safety culture research fields are strongly interconnected, drawing on largely the same science domains, with articles particularly appearing in, and drawing knowledge from the science domains “psychology, education, health”, “psychology, education, social”, and “health, nursing, medicine”. *Safety Science* is a key journal for both research domains, whereas for safety climate research, *Accident Analysis and Prevention* and *Journal of Safety Research* are important journals, and *BMC Health Services Research* and *International Journal for Quality in Health Care* are important for safety culture research.

Topic comparisons indicate that, despite the similarities, safety climate is a narrower domain of research which is primarily concerned with concepts and methods to measure safety climate, where surveys are applied in organizational contexts and their validity tested. From the viewpoint of the safety culture domain, safety climate appears to be understood as more measurement-oriented, whereas safety culture links to broader organizational aspects of assessing, assuring, and ensuring safety, such as risk assessment and safety management systems.

In both domains, by far most research and applications occur within the patient and healthcare research domains. Apart from traditional safety climate and safety culture research, which is mostly concerned with the basic concepts and theories, each domain contains applied research clusters focusing primarily on patient and healthcare. Other industrial sectors which have more recently emerged in safety climate and/or safety culture research include the construction and process industries, with the food processing industries representing the most recent subarea of activity. Not all these industries, however, focus equally on safety climate and safety culture.

Furthermore, highly cited articles are identified, which are the key knowledge bases for each subdomain. Here, it is found that despite the large body of literature on both concepts, only a relatively small number of articles are highly cited in each domain, signaling that these can be considered the core knowledge units for understanding the respective domains. In addition, from the perspective of highly cited articles, a significant overlap between the two domains was found, whereas the analysis confirmed that impactful safety climate research comparatively focuses more on aspects related to measurement, compared with safety culture research.

Finally, a number of future research directions were identified. For instance, exploring the concepts more thoroughly in other industrial or public sectors could provide additional insights, given their differing professional, organizational, and regulatory contexts. Given the dominance of research from Western countries, more theoretical and empirical work focusing on the influence of national culture is also recommended for future research.

## Figures and Tables

**Table 1:** Brief statistics of the datasets.

Dataset	Duration	Records	Articles	Reviews	Authors	Journals	Keywords
#1 Safety climate	1980–2018	492	478	14	1930	154	7717
#2 Safety culture	1991–2018	649	622	27	2545	273	7165

Note: Authors = number of authors, Journals = number of different source titles, Keywords = number of keywords.

**Table 2:** Top 10 most productive countries/regions in safety climate and safety culture research.

Safety Climate				Safety Culture			
Country/Region	Records	TNC	CPP	Country/Region	Records	TNC	CPP
USA	179	6985	39.02	USA	174	3902	22.43
Australia	84	3501	41.68	UK	74	3101	41.91
UK	51	3491	68.45	Australia	37	1038	28.05
China	41	1179	28.76	Netherlands	37	1644	44.43
Israel	26	3690	141.92	China	33	594	18.00
Canada	23	1328	57.74	Canada	31	489	15.77
Norway	20	466	23.30	Spain	25	286	11.44
Taiwan, China	20	368	18.40	Norway	23	350	15.22
Germany	17	396	23.29	Brazil	22	104	4.73
Netherlands	12	377	31.42	Germany	22	107	4.86
Spain	12	527	43.92	Taiwan, China	22	306	13.91
Sweden	12	311	25.92				

Note: TNC = total number of citations, CPP = citations per paper.

**Table 3:** Top 10 most productive institutions in safety climate and safety culture research.

Safety Climate				Safety Culture			
Institution	Records	TNC	CPP	Institution	Records	TNC	CPP
Harvard University	23	986	42.9	University of Pittsburgh	14	550	39.3
Liberty Mutual Research Institute for Safety	19	827	43.5	VU Amsterdam Medical Center	10	290	29.0
Technion–Israel Institute of Technology	18	3601	200.1	Johns Hopkins University	9	547	60.8
University of South Australia	17	804	47.3	University of Michigan	9	302	33.6
Queensland University of Technology	16	219	13.7	VU Amsterdam	9	106	11.8
Stanford University	13	786	60.5	Griffith University	8	544	68.0
University of Aberdeen	12	1697	141.4	Netherlands Institute for Health Services Research	8	206	25.8
Hong Kong Polytechnic University	11	119	10.8	University of Manchester	8	357	44.6
University of Manchester	11	749	68.1	University of Oslo	8	135	16.9
University of Bergen	10	164	16.4	Westat Corporation	8	490	61.3
University of Connecticut	10	243	24.3				

Note: TNC = total number of citations, CPP = citations per paper.

**Table 4:** Citation trends in safety culture and safety climate at a domain level.

Dataset	Journals Citing Domain	Journals Cited Domain	Z-Score
#1 Safety climate	Psychology, education, health	Psychology, education, social	6.61
	Psychology, education, health	Health, nursing, medicine	2.57
	Medicine, medical, clinical	Health, nursing, medicine	2.37
#2 Safety culture	Medicine, medical, clinical	Health, nursing, medicine	6.93
	Psychology, education, health	Health, nursing, medicine	5.06
	Psychology, education, health	Psychology, education, social	3.76

**Table 5:** Citing and cited journals for safety climate and safety culture publications.

	Safety Climate	Safety Culture
Citing journals	Safety Science (58) Accident Analysis and Prevention (44) Journal of Safety Research (30) BMJ Quality & Safety (14) Journal of Applied Psychology (14)	Safety Science (59) BMC Health Services Research (35) International Journal for Quality in Health Care (23) BMJ Quality & Safety (22) Journal of Patient Safety (17)
Cited journals	Journal of Applied Psychology (1983) Safety Science (1805) Journal of Safety Research (996) Accident Analysis and Prevention (870) Journal of Occupational Health Psychology (526)	Safety Science (1389) Quality & Safety in Health Care (916) BMC Health Services Research (593) International Journal for Quality in Health Care (335) Journal of Safety Research (334)

**Table 6:** Distribution of keywords for safety climate and safety culture research (minimum occurrence ≥ 5).

Safety Climate			Safety Culture		
Keyword	Occurrences	Avg. Pub. Year	Keyword	Occurrences	Avg. Pub. Year
*Safety climate*	244	2012.10	*Safety culture*	235	2012.11
*Patient safety*	57	2014.26	*Patient safety*	154	2014.24
*Safety culture*	46	2011.85	Patient safety culture	78	2015.14
Psychosocial safety climate	27	2015.19	*Organizational culture*	52	2012.58
*Safety performance*	27	2010.74	*Safety*	50	2011.18
Occupational safety	19	2012.21	*Safety management*	38	2012.47
Organizational climate	16	2009.81	*Safety climate*	34	2010.79
*Safety*	16	2013.69	*Culture*	33	2013.39
Safety behavior	15	2013.33	*Hospital*	24	2015.38
*Safety management*	15	2010.27	*Survey*	23	2014.00
*Survey*	15	2013.00	Medical error	18	2012.33
*Nurses*	14	2012.43	*Nurses*	17	2015.06
Structural equation modeling	12	2012.75	Nursing	16	2013.88
Injuries	11	2013.27	Adverse events	15	2014.00
Job satisfaction	11	2014.27	Nursing home	15	2012.13
*Organizational culture*	10	2015.00	*Quality of health care*	15	2015.40
Confirmatory factor analysis	9	2013.22	Risk management	15	2012.07
Factor analysis	9	2009.78	HOSPSC	13	2014.15
*Hospital*	9	2013.22	*Questionnaire*	12	2014.67
Multilevel	9	2013.56	*Safety attitudes*	12	2008.25
*Communication*	8	2013.88	*Safety performance*	11	2012.45
*Construction*	8	2012.25	*Primary care*	10	2015.40
Psychological safety climate	8	2014.75	Quality improvement	10	2015.50
Safety compliance	8	2012.88	Food safety culture	9	2016.44
Construction safety	7	2015.29	*Healthcare*	9	2013.00
*Healthcare*	7	2012.71	Validation	9	2011.78
*Leadership*	7	2012.57	Organizations	8	2011.00
Risk perception	7	2012.00	Primary health care	8	2016.50
Safety perceptions	7	2012.43	Safety management system	8	2007.13
Construction industry	6	2015.83	Attitudes	7	2010.14
Patient safety climate	6	2014.17	*Communication*	7	2013.43
*Primary care*	6	2015.17	*Construction*	6	2014.17
*Questionnaire*	6	2012.33	Food safety	6	2013.33
*Safety attitudes*	6	2007.67	Healthcare organization	6	2015.50
Work stress	6	2012.50	Human factors	6	2010.33
Workplace injury	6	2011.33	Intensive care unit	6	2015.00
Burnout	5	2016.60	*Leadership*	6	2012.67
Climate	5	2013.80	Occupational health and safety	6	2011.50
*Culture*	5	2014.60	Psychometric	6	2015.83
Depression	5	2014.20	Safety attitude questionnaire	6	2014.83
Emotional exhaustion	5	2015.20	Checklist	5	2014.60
Exploratory factor analysis	5	2014.80	Critical care	5	2012.00
Immigrant workers	5	2014.00	Long-term care	5	2013.80
Measurement equivalence	5	2014.20	Management	5	2013.20
Organizational safety climate	5	2013.00	Nuclear industry	5	2012.00
Psychosocial risk	5	2016.00	Nuclear power plant	5	2013.60
*Quality of health care*	5	2014.20	Patient	5	2014.60
Safety participation	5	2010.40	——	——	——
Theory of planned behavior	5	2012.60	——	——	——

Note: Occurrences = number of papers. Avg. Pub. Year is the average publication year of a certain keyword in all papers. Keywords in italics appear in both sides of the table.

## Supplementary Materials

The following supporting information can be downloaded at: https://www.mdpi.com/article/10.3390/ijerph19020813/s1, Table S1. Highest cited papers on safety climate (ranked by publication year), Table S2. Highest cited papers on safety culture (ranked by publication year), Table S3. Process safety culture and food safety culture papers in citation network (ranked by publication year).
