# Supplementary material for: Conceptualizing the Contextual Dynamics of Safety Climate and Safety Culture Research: A Comparative Scientometric Analysis
Source: Int J Environ Res Public Health. 2022 Jan 12;19(2):813. doi: 10.3390/ijerph19020813 (PMC8775979; doi:10.3390/ijerph19020813)
Supplement: Supplementary file 1 [file ijerph-19-00813-s001.zip › ijerph-1458155-supplementary.pdf]

Supplemental Material Table

Table S1. Highest cited papers on safety climate (ranked by publication year).

Authors	Title	Journal	Year	Cit_Score
Zohar, D	Safety climate in industrial-organizations - theoretical and applied implications	*Journal of Applied Psychology*	1980	247
Brown, Rl; Holmes, H	The use of a factor-analytic procedure for assessing the validity of an employee safety climate model	*Accident Analysis and Prevention*	1986	67
Dedobbeleer, N; Beland, F	A Safety Climate Measure for Construction Sites	Journal of Safety Research	1991	87
Niskanen, T	Safety climate in the road administration	Safety Science	1994	34
Coyle, Ir; Sleeman, Sd; Adams, N	Safety climate	Journal of Safety Research	1995	53
Diaz, Ri; Cabrera, Dd	Safety climate and attitude as evaluation measures of organizational safety	Accident Analysis and Prevention	1997	33
Williamson, Am; Feyer, Am; Cairns, D; Biancotti, D	The development of a measure of safety climate: the role of safety perceptions and attitudes	Safety Science	1997	40
Hofmann, Da; Stetzer, A	The role of safety climate and communication in accident interpretation: implications for learning from negative events	Academy of Management Journal	1998	27
Mearns, K; Flin, R; Gordon, R; Fleming, M	Measuring safety climate on offshore installations	Work and Stress	1998	47
Cheyne, A; Cox, S; Oliver, A; Tomas, Jm	Modelling safety climate in the prediction of levels of safety activity	Work and Stress	1998	54
Varonen, U; Mattila, M	The safety climate and its relationship to safety practices, safety of the work environment and occupational accidents in eight wood-processing companies	Accident Analysis and Prevention	2000	40
Zohar, D	A group-level model of safety climate: testing the effect of group climate on microaccidents in manufacturing jobs	Journal of Applied Psychology	2000	141
Gershon, Rrm; Karkashian, Cd; Grosch, Jw; Murphy, Lr; Escamilla-Cejudo, A; Flanagan, Pa; Bernacki, E; Kasting, C; Martin, L	Hospital safety climate and its relationship with safe work practices and workplace exposure incidents	American Journal of Infection Control	2000	44
Neal, A; Griffin, Ma; Hart, Pm	The impact of organizational climate on safety climate and individual behavior	Safety Science	2000	123
Flin, R; Mearns, K; O'connor, P; Bryden, R	Measuring safety climate: identifying the common features	Safety Science	2000	149
Glendon, Ai; Litherland, Dk	Safety climate factors, group differences and safety behaviour in road construction	Safety Science	2001	64
Mearns, K; Whitaker, Sm; Flin, R	Benchmarking safety climate in hazardous environments: a longitudinal, interorganizational approach	Risk Analysis	2001	25
Mohamed, S	Safety climate in construction site environments	Journal of Construction Engineering And Management-ASCE	2002	40
Gillen, M; Baltz, D; Gassel, M; Kirsch, L; Vaccaro, D	Perceived safety climate, job demands, and coworker support among union and nonunion injured construction workers	Journal of Safety Research	2002	62

Authors	Title	Journal	Year	Cit_Score
Zohar, D	The effects of leadership dimensions, safety climate, and assigned priorities on minor injuries in work groups	Journal of Organizational Behavior	2002	56
Mearns, K; Whitaker, Sm; Flin, R	Safety climate, safety management practice and safety performance in offshore environments	Safety Science	2003	68
Hofmann, Da; Morgeson, Fp; Gerras, Sj	Climate as a moderator of the relationship between leader-member exchange and content specific citizenship: safety climate as an exemplar	Journal of Applied Psychology	2003	48
Siu, Ol; Phillips, Dr; Leung, Tw	Safety climate and safety performance among construction workers in hong kong - the role of psychological strains as mediators	Accident Analysis and Prevention	2004	59
Dejoy, Dm; Schaffer, Bs; Wilson, Mg; Vandenberg, Rj; Butts, Mm	Creating safer workplaces: assessing the determinants and role of safety climate	Journal of Safety Research	2004	49
Seo, Dc; Torabi, Mr; Blair, Eh; Ellis, Nt	A cross-validation of safety climate scale using confirmatory factor analytic approach	Journal of Safety Research	2004	39
Cooper, Md; Phillips, Ra	Exploratory analysis of the safety climate and safety behavior relationship	Journal of Safety Research	2004	67
Katz-Navon, T; Naveh, E; Stern, Z	Safety Climate in Health Care Organizations: A Multidimensional Approach	Academy of Management Journal	2005	25
Colla, Jb; Bracken, Ac; Kinney, Lm; Weeks, Wb	Measuring patient safety climate: a review of surveys	Quality & Safety in Health Care	2005	52
Zohar, D; Luria, G	A multilevel model of safety climate: cross-level relationships between organization and group-level climates	Journal of Applied Psychology	2005	128
Hofmann, Da; Mark, B	An investigation of the relationship between safety climate and medication errors as well as other nurse and patient outcomes	Personnel Psychology	2006	42
Huang, Yh; Ho, M; Smith, Gs; Chen, Py; Mith, S	Safety climate and self-reported injury: assessing the mediating role of employee safety control	Accident Analysis and Prevention	2006	41
Neal, A; Griffin, Ma	A study of the lagged relationships among safety climate, safety motivation, safety behavior, and accidents at the individual and group levels	Journal of Applied Psychology	2006	110
Clarke, S	The relationship between safety climate and safety performance: a meta-analytic review	Journal of Occupational Health Psychology	2006	95
Fang, Dp; Chen, Y; Wong, L	Safety climate in construction industry: a case study in hong kong	Journal of Construction Engineering and Management-ASCE	2006	32
Wallace, Jc; Popp, E; Mondore, S	Safety climate as a mediator between foundation climates and occupational accidents: a group-level investigation	Journal of Applied Psychology	2006	25
Flin, R; Burns, C; Mearns, K; Yule, S; Robertson, Em	Measuring safety climate in health care	Quality & Safety in Health Care	2006	53
Johnson, Se	The predictive validity of safety climate	Journal of Safety Research	2007	38
Singer, S; Meterko, M; Baker, L; Gaba, D; Falwell, A; Rosen, A	Workforce perceptions of hospital safety culture: development and validation of the patient safety climate in healthcare organizations survey	Health Services Research	2007	31
Hahn, Se; Murphy, Lr	A short scale for measuring safety climate	Safety Science	2008	27
Dov, Z	Safety climate and beyond: a multi-level multi-climate framework	Safety Science	2008	34
Pousette, A; Larsson, S; Torner, M	Safety climate cross-validation, strength and prediction of safety behaviour	Safety Science	2008	30
Singer, S; Lin, S; Falwell, A; Gaba, D; Baker, L	Relationship of safety climate and safety performance in hospitals	Health Services Research	2009	31

Authors	Title	Journal	Year	Cit_Score
Clarke, S	An integrative model of safety climate: linking psychological climate and work attitudes to individual safety outcomes using meta-analysis	Journal of Occupational and Organizational Psychology	2010	41
Dollard, Mf; Bakker, Ab	Psychosocial safety climate as a precursor to conducive work environments, psychological health problems, and employee engagement	Journal of Occupational and Organizational Psychology	2010	35
Hall, Gb; Dollard, Mf; Coward, J	Psychosocial safety climate: development of the psc-12	International Journal of Stress Management	2010	28
Zohar, D	Thirty years of safety climate research: reflections and future directions	Accident Analysis and Prevention	2010	104
Beus, Jm; Payne, Sc; Bergman, Me; Arthur, W	Safety climate and injuries: an examination of theoretical and empirical relationships	Journal of Applied Psychology	2010	63
Law, R; Dollard, Mf; Tuckey, Mr; Dormann, C	Psychosocial safety climate as a lead indicator of workplace bullying and harassment, job resources, psychological health and employee engagement	Accident Analysis and Prevention	2011	28
Idris, Ma; Dollard, Mf; Coward, J; Dormann, C	Psychosocial safety climate: conceptual distinctiveness and effect on job demands and worker psychological health	Safety Science	2012	27
Huang, Yh; Zohar, D; Robertson, Mm; Garabet, A; Lee, J; Murphy, La	Development and validation of safety climate scales for lone workers using truck drivers as exemplar	Transportation Research Part F-Traffic Psychology and Behaviour	2013	30

Table S2. Highest cited papers on safety culture (ranked by publication year).

Authors	Title	Journal	Year	Cit_Score
Pidgeon, Nf	Safety culture and risk management in organizations	*Journal of Cross-Cultural Psychology*	1991	28
Ostrom, L; Wilhelmsen, C; Kaplan, B	Assessing safety culture	*Nuclear Safety*	1993	16
Cox, S; Flin, R	Safety culture: philosopher's stone or man of straw?	Work and Stress	1998	42
Pidgeon, N	Safety culture: key theoretical issues	Work and Stress	1998	27
Lee, T	Assessment of safety culture at a nuclear reprocessing plant	Work and Stress	1998	22
Mearns, Kj; Flin, R	Assessing the state of organizational safety culture or climate?	Current Psychology	1999	14
Clarke, S	Perceptions of organizational safety: implications for the development of safety culture	Journal of Organizational Behavior	1999	35
Cooper, Md	Towards a model of safety culture	Safety Science	2000	88
Grote, G; Kunzler, C	Diagnosis of safety culture in safety management audits	Safety Science	2000	27
Lee, T; Harrison, K	Assessing safety culture in nuclear power stations	Safety Science	2000	28
Cox, Sj; Cheyne, Ajt	Assessing safety culture in offshore environments	Safety Science	2000	27
Guldenmund, Fw	The nature of safety culture: a review of theory and research	Safety Science	2000	108
Glendon, Ai; Stanton, Na	Perspectives on Safety Culture	Safety Science	2000	36
Sorensen, Jn	Safety culture: a survey of the state-of-the-art	Reliability Engineering & System Safety	2002	25
Nieva, Vf; Sorra, J	Safety culture assessment: a tool for improving patient safety in healthcare organizations	Quality & Safety in Health Care	2003	75
Ginsburg, L; Norton, Pg; Casebeer, A; Lewis, S	An educational intervention to enhance nurse leaders' perceptions of patient safety culture	Health Services Research	2005	14
Pronovost, P; Sexton, B	Assessing safety culture: guidelines and recommendations	Quality & Safety in Health Care	2005	50
Handler, Sm; Castle, Ng; Studenski, Sa; Perera, S; Fridsma, Db; Nace, Da; T Hanlon, J	Patient safety culture assessment in the nursing home	Quality & Safety in Health Care	2006	18
Parker, D; Lawrie, M; Hudson, P	A framework for understanding the development of organisational safety culture	Safety Science	2006	30
Singer, S; Meterko, M; Baker, L; Gaba, D; Falwell, A; Rosen, A	Workforce perceptions of hospital safety culture: development and validation of the patient safety climate in healthcare organizations survey	Health Services Research	2007	15
Choudhry, Ra; Fang, Dp; Mohamed, S	The nature of safety culture: a survey of the state-of-the-art	*Safety Science*	2007	37
Kirk, S; Parker, D; Claridge, T; Esmail, A; Marshall, M	Patient safety culture in primary care: developing a theoretical framework for practical use	Quality & Safety in Health Care	2007	31
Flin, R	Measuring safety culture in healthcare: a case for accurate diagnosis	Safety Science	2007	35
Guldenmund, Fw	The use of questionnaires in safety culture research - an evaluation	Safety Science	2007	32
Huang, Dt; Clermont, G; Sexton, Jb; Karlo, Ca; Miller, Rg; Weissfeld, La; Rowan, Km; Angus, Dc	Perceptions of safety culture vary across the intensive care units of a single institution	Critical Care Medicine	2007	31
Modak, I; Sexton, Jb; Lux, Tr; Helmreich, Rl; Thomas, Ej	Measuring safety culture in the ambulatory setting: the safety attitudes questionnaire-ambulatory version	Journal of General Internal Medicine	2007	21
Fernandez-Muniz, B; Montes-Peon, Jm; Vazquez-Ordas, Cj	Safety culture: analysis of the causal relationships between its key dimensions	Journal of Safety Research	2007	15

Authors	Title	Journal	Year	Cit_Score
Vogus, Tj; Sutcliffe, Km	The safety organizing scale - development and validation of a behavioral measure of safety culture in hospital nursing units	Medical Care	2007	15
Smits, M; Christiaans-Dingelhoff, I; Wagner, C; Van Der Wal, G; Groenewegen, Pp	The psychometric properties of the 'hospital survey on patient safety culture' in dutch hospitals	BMC Health Services Research	2008	52
Bodur, S; Filiz, E	A survey on patient safety culture in primary healthcare services in turkey	International Journal for Quality in Health Care	2009	32
Smits, M; Wagner, C; Spreeuwenberg, P; Van Der Wal, G; Groenewegen, Pp	Measuring patient safety culture: an assessment of the clustering of responses at unit level and hospital level	Quality & Safety in Health Care	2009	27
Wagner, Lm; Capezuti, E; Rice, Jc	Nurses' perceptions of safety culture in long-term care settings	Journal of Nursing Scholarship	2009	15
Pfeiffer, Y; Manser, T	Development of the german version of the hospital survey on patient safety culture: dimensionality and psychometric properties	Safety Science	2010	25
El-Jardali, F; Jaafar, M; Dimassi, H; Jamal, D; Hamdan, R	The current state of patient safety culture in lebanese hospitals: a study at baseline	International Journal for Quality in Health Care	2010	36
Alahmadi, Ha	Assessment of patient safety culture in saudi arabian hospitals	Quality & Safety in Health Care	2010	17
Waterson, P; Griffiths, P; Stride, C; Murphy, J; Hignett, S	Psychometric properties of the hospital survey on patient safety culture: findings from the uk	Quality & Safety in Health Care	2010	22
Lee, Wc; Wung, Hy; Liao, Hh; Lo, Cm; Chang, Fl; Wang, Pc; Fan, A; Chen, Hh; Yang, Hc; Hou, Sm	Hospital safety culture in taiwan: a nationwide survey using chinese version safety attitude questionnaire	BMC Health Services Research	2010	18
Sorra, Js; Dyer, N	Multilevel psychometric properties of the ahrq hospital survey on patient safety culture	BMC Health Services Research	2010	64
Chen, Ic; Li, Hh	Measuring patient safety culture in taiwan using the hospital survey on patient safety culture (hsopsc)	BMC Health Services Research	2010	36
Huang, Dt; Clermont, G; Kong, L; Weissfeld, La; Sexton, Jb; Rowan, Km; Angus, Dc	Intensive care unit safety culture and outcomes: a us multicenter study(dagger)	International Journal for Quality in Health Care	2010	26
Bodur, S; Filiz, E	Validity and reliability of turkish version of "hospital survey on patient safety culture" and perception of patient safety in public hospitals in turkey	BMC Health Services Research	2010	30
Sammer, Ce; Lykens, K; Singh, Kp; Mains, Da; Lackan, Na	What is patient safety culture? A review of the literature	Journal of Nursing Scholarship	2010	42
Halligan, M; Zecevic, A	Safety culture in healthcare: a review of concepts, dimensions, measures and progress	BMJ Quality & Safety	2011	29
Sarac, C; Flin, R; Mearns, K; Jackson, J	Hospital survey on patient safety culture: psychometric analysis on a scottish sample	BMJ Quality & Safety	2011	17
El-Jardali, F; Dimassi, H; Jamal, D; Jaafar, M; Hemadeh, N	Predictors and outcomes of patient safety culture in hospitals	BMC Health Services Research	2011	25
Ito, S; Seto, K; Kigawa, M; Fujita, S; Hasegawa, T; Hasegawa, T	Development and applicability of hospital survey on patient safety culture (hsops) in japan	BMC Health Services Research	2011	15
Hamdan, M; Saleem, Aa	Assessment of patient safety culture in palestinian public hospitals	International Journal for Quality in Health Care	2013	15
Morello, Rt; Lowthian, Ja; Barker, Al; Mcginnes, R; Dunt, D; Brand, C	Strategies for improving patient safety culture in hospitals: a systematic review	BMJ Quality & Safety	2013	29
Wagner, C; Smits, M; Sorra, J; Huang, Cc	Assessing patient safety culture in hospitals across countries	International Journal for Quality in Health Care	2013	23
Nie, Yl; Mao, Xy; Cui, H; He, Sh; Li, J; Zhang, Mm	Hospital survey on patient safety culture in china	BMC Health Services Research	2013	19

Table S3. Process safety culture and food safety culture papers in citation network (ranked by publication year).

Authors	Title	Source	Year	Cit_Score
Process Safety Culture				
Frank, Wl	Process safety culture in the ccps risk-based process safety model	Process Safety Progress	2007	6
Sutton, Is	Use root cause analysis to understand and improve process safety culture	Process Safety Progress	2008	2
Kao, Cs; Lai, Wh; Chuang, Tf; Lee, Jc	Safety culture factors, group differences, and risk perception in five petrochemical plants	Process Safety Progress	2008	5
Mckay, M; Lacoursiere, Jp	Development of a process safety culture of chemical engineers	Process Safety Progress	2008	2
Shirali, G; Mohammadfam, I; Motamedzade, M; Ebrahimipour, V; Moghimbeigi, A	Assessing resilience engineering based on safety culture and managerial factors	Process Safety Progress	2012	2
Wang, Ch; Liu, Yj	The dimensions and analysis of safety culture	Process Safety Progress	2012	0
Forest, Jj	How to evaluate process safety culture	Process Safety Progress	2012	0
Hendershot, Dc	Process safety management-you can't get it right without a good safety culture	Process Safety Progress	2012	0
Louvar, Jf	How to communicate to create a safety culture and improve psm results	Process Safety Progress	2013	0
O'neal, Aem; Clavaud, O	Process safety culture: a creative potential approach to extract more value from risk assessments	Journal of Loss Prevention in The Process Industries	2016	0
Food Safety Culture				
Griffith, Cj	Do businesses get the food poisoning they deserve? The importance of food safety culture	British Food Journal	2010	6
Griffith, Cj; Livesey, Km; Clayton, Da	Food safety culture: the evolution of an emerging risk factor?	British Food Journal	2010	7
Griffith, Cj; Livesey, Km; Clayton, D	The assessment of food safety culture	British Food Journal	2010	10
Powell, Da; Jacob, Cj; Chapman, Bj	Enhancing food safety culture to reduce rates of foodborne illness	Food Control	2011	9
Seward, S; Dobmeier, N; Baron, M	Assessing the food safety culture of a manufacturing facility	Food Technology	2012	1
Fatimah, Uzau; Strohbehn, Ch; Arendt, Sw	An empirical investigation of food safety culture in onsite foodservice operations	Food Control	2014	4
Jespersen, L; Griffiths, M; Maclaurin, T; Chapman, B; Wallace, Ca	Measurement of food safety culture using survey and maturity profiling tools	Food Control	2016	7
Nyarugwe, Sp; Linnemann, A; Hofstede, Gj; Fogliano, V; Luning, Pa	Determinants for conducting food safety culture research	Trends in Food Science & Technology	2016	3
Baur, P; Getz, C; Sowerwine, J	Contradictions, consequences and the human toll of food safety culture	Agriculture and Human Values	2017	0
Griffith, Cj; Jackson, Lm; Lues, R	The food safety culture in a large south african food service complex perspectives on a case study	British Food Journal	2017	0
Jespersen, L; Griffiths, M; Wallace, Ca	Comparative analysis of existing food safety culture evaluation systems	Food Control	2017	2
Jespersen, L; Maclaurin, T; Vlerick, P	Development and validation of a scale to capture social desirability in food safety culture	Food Control	2017	1
Nayak, R; Waterson, P	The assessment of food safety culture: an investigation of current challenges, barriers and future opportunities within the food industry	Food Control	2017	0

Jespersen, L; Wallace, Ca	Triangulation and the importance of establishing valid methods for food safety culture evaluation	Food Research International	2017	0
De Boeck, E; Mortier, Av; Jacxsens, L; Dequidt, L; Vlerick, P	Towards an extended food safety culture model: studying the moderating role of burnout and jobstress, the mediating role of food safety knowledge and motivation in the relation between food safety climate and food safety behavior	Trends in Food Science & Technology	2017	1
Nyarugwe, Sp; Linnemann, A; Nyanga, Lk; Fogliano, V; Luning, Pa	Food safety culture assessment using a comprehensive mixed-methods approach: a comparative study in dairy processing organisations in an emerging economy	Food Control	2018	0
